# MetaMerge: scaling up genome-scale metabolic reconstructions with application to *Mycobacterium tuberculosis*

**DOI:** 10.1186/gb-2012-13-1-r6

**Published:** 2012-01-31

**Authors:** Leonid Chindelevitch, Sarah Stanley, Deborah Hung, Aviv Regev, Bonnie Berger

**Affiliations:** 1 Department of Mathematics, Massachusetts Institute of Technology, 77 Massachusetts Avenue, Cambridge, MA 02139, USA; 2 Computer Science and Artificial Intelligence Laboratory, Massachusetts Institute of Technology, 32 Vassar Street, Cambridge, MA 02139, USA; 3 Department of Molecular Biology, Massachusetts General Hospital, Simches Research Center, 185 Cambridge Street, Boston, MA 02114, USA; 4 Broad Institute of MIT and Harvard, 7 Cambridge Center, Cambridge, MA 02142, USA; 5 Department of Biology, Massachusetts Institute of Technology, 77 Massachusetts Avenue, Cambridge, MA 02139, USA; 6 Howard Hughes Medical Institute, 4000 Jones Bridge Road, Chevy Chase, MD 20815, USA

## Abstract

Reconstructed models of metabolic networks are widely used for studying metabolism in various organisms. Many different reconstructions of the same organism often exist concurrently, forcing researchers to choose one of them at the exclusion of the others. We describe MetaMerge, an algorithm for semi-automatically reconciling a pair of existing metabolic network reconstructions into a single metabolic network model. We use MetaMerge to combine two published metabolic networks for *Mycobacterium tuberculosis *into a single network, which allows many reactions that could not be active in the individual models to become active, and predicts essential genes with a higher positive predictive value.

## Background

Due to the recent explosion in the number of sequenced genomes of unicellular organisms, it has become possible to systematically analyze their biochemical functionality. Analysis of reconstructed metabolic networks - most commonly through constraint-based approaches, such as flux-balance analysis [1] - has become a tool of choice for systematically and computationally studying the biochemical processes relevant to these organisms' metabolism [2,3]. Despite certain limitations of the analysis methods (for example, capturing only quasi-steady-state properties) and the reconstructed models (for example, lacking information on reaction directionality under physiological conditions), this approach has elucidated important features of the metabolism of diverse organisms [4], identified essential genes [5], and proposed potential metabolic drug target candidates [6].

Metabolic network models are typically constructed based on a genome's annotated sequence and existing biochemical knowledge [7]. Genome-scale metabolic network reconstruction is a labor-intensive process that relies on extensive searches in the scientific literature and in databases, such as the Kyoto Encyclopedia of Genes and Genomes (KEGG) [8], to determine the set of reactions available to a given organism. The reconstruction process consists of three stages: first, the genome annotation is used to identify candidate metabolic reactions; second, reactions are localized in the cell, assigned directionality whenever possible, and associated with specific enzymes; and finally, exchange, transport and growth reactions are added to the model. This process has been successfully applied to many organisms, including bacterial pathogens such as *Bacillus subtilis*, *Escherichia coli*, *Haemophilus influenzae*, *Mycobacterium tuberculosis *and *Staphylococcus aureus*. When experimental data are available, the reconstructed metabolic model is often further refined, as was done for *Acinetobacter baylyi *[9] and for *Saccharomyces cerevisiae *[10].

The complexity of the reconstruction process and the large number of literature sources on metabolism have often led to the generation of multiple metabolic models for the same organism. Indeed, the 59 metabolic networks currently available [11] only represent 39 distinct organisms. Thus, researchers typically must consider each of the available reconstructions individually.

To date, very few works have addressed the problem of reconciling different reconstructions for the same organism. Recent studies have attempted to align metabolic networks of two different organisms [12]. However, such alignment approaches can only identify a common part of the two networks considered, and the goal of aligning two networks is usually to find common pathways or understand the evolutionary history of metabolism. On the other hand, the goal of combining (reconciling) a pair of networks is to preserve the complete functionality of each of the original networks while combining the information contained in them. We believe that the problem of network reconciliation is of independent interest because its solution enables researchers to access an integrated metabolic model without losing the functionality of either of the available models for a given organism. Importantly, comparing between two different reconstructions is challenging since the reviewed bodies of literature may not be the same, the naming conventions often differ between different models, and the extent of annotation typically varies from one model to another.

These discrepancies are illustrated in a comparison of the two main existing metabolic networks published for *M. tuberculosis *by Beste *et al*. [13] and by Jamshidi and Palsson [14]. (An earlier model by Raman *et al*. [15] covers only the mycolic acid pathway, which is included in one of the genome-scale models [13], and hence will not be considered here.) First, although both models were prepared based on extensive literature curation, they do not cover the same set of publications related to the metabolism of *M. tuberculosis*: only 21 articles are shared between the 100 articles referenced by Beste *et al*. [13] and the 141 referenced by Jamshidi and Palsson [14]. Second, the models are annotated using different convention systems, rendering comparison difficult, as metabolites are named according to different nomenclatures, the reactions are classified into different pathways, and the combinations of genes necessary for each reaction are formatted differently.

Here, we present MetaMerge, an algorithm for reconciling two or more metabolic networks. MetaMerge takes as input two network models and produces a unified network that conserves the functionality of both of the original networks, while providing additional functionality whenever the two networks complement one another. MetaMerge can act without any supervision, although some minimal supervision results in a more reliable model. We applied MetaMerge to the two existing networks for *M. tuberculosis*, a pathogen whose metabolism is poorly understood, especially during infection [13,14]. Although each network individually covers a large fraction of the available metabolic reactions, we find here that the two networks can be combined in a synergistic fashion. In particular, the combined model has over 60% fewer 'blocked' reactions (that is, reactions that cannot have any flux through them at steady state) than the two individual models, and predicts essential genes with a higher positive predictive value than the individual models. We further use the model to nominate a shortlist of metabolic genes whose perturbation may mimic the metabolic effect of several known anti-tubercular drugs.

## Results

### The MetaMerge algorithm for reconciling metabolic models

**Figure 1:**
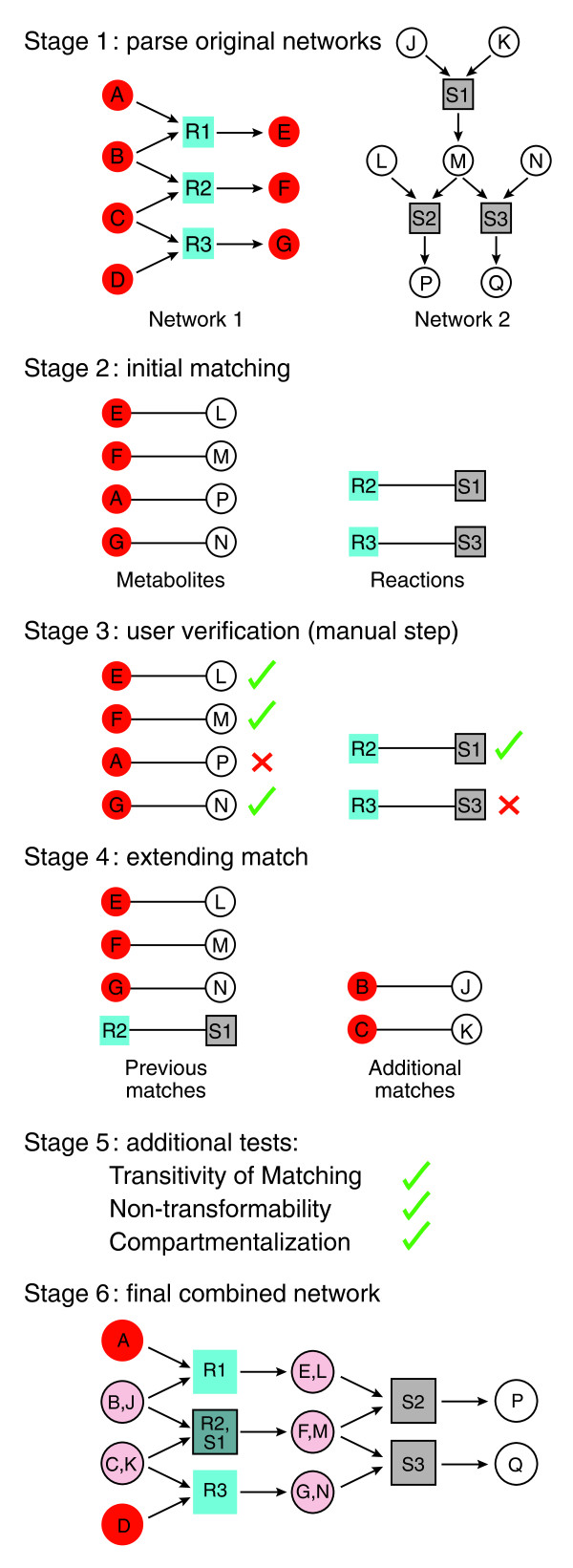
**Flow chart for the MetaMerge algorithm**. Shown is a flow chart of the six stages of the MetaMerge algorithm. Stage 1: the two models to be merged are parsed and the features for metabolites and reactions are prepared. Stage 2: an initial matching of reactions and metabolites is created. Stage 3: the user is asked to confirm the newly matched reactions and metabolites (optional step). Stage 4: the newly matched metabolites are used to extend the current matching of reactions and metabolites. Stages 3 and 4 are repeated until convergence. Stage 5: the matching of metabolites is checked to ensure transitivity and non-transformability. Stage 6: the merger is performed and the resulting network is output in the desired format. Circles indicate metabolites (red, model 1; white, model 2; pink, combined); squares indicate reactions (turquoise, model 1; brown, model 2; dark green, combined). The mini-networks were created using the Cytoscape software [34], version 2.7.0.

We developed MetaMerge (Figure 1), a semi-automatic algorithm for merging two metabolic networks. MetaMerge was originally intended to assist the routine parts of a manual network reconciliation process; however, due to the extreme complexity of the manual reconciliation process (which took the authors several months to complete while producing a substantially less satisfying result in terms of the criteria described below), MetaMerge was later expanded to cover all the phases of the reconciliation process, requiring minimal user input.

MetaMerge consists of six stages: stage 1, model parsing and pre-processing; stage 2, initial matching of reactions and metabolites between the models; stage 3, (optional) user confirmation of the matching in step 2; stage 4, iterative extension, matching additional reactions and metabolites; stage 5, checking the metabolite matching to ensure transitivity and non-transformability (two important properties described in the Materials and methods); and stage 6, merging and providing the resulting network in the desired format. We concisely describe each stage below and provide full details in the Materials and methods.

MetaMerge begins by parsing and pre-processing each model, augmenting each metabolite and reaction with several features (Figure 1, stage 1). These include the full names and molecular formulas for the metabolites and enzyme names and pathway names for the reactions. More richly annotated models may have additional features, such as standardized identifiers for the metabolites and enzymes. In less richly annotated models, MetaMerge can automatically extract these additional features by querying online databases, such as KEGG [8]. Our aim is to have standardized identifiers for as many metabolites and reactions as possible to facilitate comparison; for example, sodium bicarbonate, bicarbonate of soda, baking soda and sodium hydrogen carbonate all have the same Chemical Abstracts Service (CAS) number, 144-55-8 [16].

During the merging process (Figure 1, stages 2 to 5), MetaMerge matches entities (metabolites or reactions) between the two models based on the number of features they have in common; the more features they share, the more likely they are to be chosen as a match. Distinct types of features (for example, the CAS number and the molecular formula) are weighted equally, and hence have the same impact on the matching; however, user-defined weights reflecting the confidence for each type of matching feature can be accommodated. Matches are not necessarily one-to-one. In some cases, one metabolite in one model is subcategorized into several metabolites in another model, resulting in one-to-many matches. For example, in the two *M. tuberculosis *models trehalose dimycolate is denoted as TREHALOSEDIMYCOLATE in one model [13], but is subdivided into tdm1 through tdm4 in the other [14], depending on the type of mycolates that get attached to the trehalose.

Since not all matches are one-to-one, MetaMerge introduces a matching matrix, to which it applies graph-theoretic algorithms. The matching matrix is a binary matrix with a row for each entity from one model and a column for each entity from the other model, where an entity refers to either a metabolite or a reaction. The matrix contains a 1 in those entries that correspond to a match, and a 0 elsewhere. The matrix format ensures that the merging works symmetrically for the two models, and that the result would not change if the two models were to be considered in the opposite order.

The matching matrices (one for the metabolites and one for the reactions) are initialized in stage 2, based on the number of features that the entities have in common. The matrices are possibly cleaned up in stage 3, when the user can choose to either accept or reject any given match. Finally, they are expanded in stage 4, based on the reasoning that reactions that have almost all their metabolites in common are likely to match one another, and that the remaining metabolites in those reactions are likely to match as well. Stages 3 and 4 are then iterated until no further expansion can be proposed, or until every entity in one of the models has been matched (the latter case being unlikely due to the incomplete overlap between any pair of models).

During post-processing (Figure 1, stage 5), MetaMerge checks two conditions. The first condition ensures that the metabolites of the two models can be divided into non-overlapping classes, with no matches occurring between classes and all possible matches occurring inside a class. Each class corresponds to a single metabolite in the combined model, and with rare exceptions (such as the trehalose dimycolate example above) consists of a single metabolite from each original model. The second condition ensures that no two metabolites in the same class appear on different sides of the same reaction. If the metabolites in the models are divided into cellular compartments, MetaMerge checks a third condition that ensures that metabolites in a certain compartment in one of the models are matched only to metabolites in the corresponding compartment in the other model. Any violations of these conditions, though rare, are identified automatically, but must be repaired manually.

### Preparation of the combined model

In the final stage (Figure 1, stage 6), MetaMerge prepares the combined model from the matching matrix for metabolites. MetaMerge's goal is to ensure that the combined model preserves the full functionality of each individual model, while allowing for new, synergistic functionality not present in the individual models. Because constraint-based formalisms [1], such as flux balance analysis, are the most common way of analyzing reconstructed metabolic models, MetaMerge uses an approach that ensures that mass-balance constraints on each metabolite in the combined model are no more stringent than they were in the initial models, and possibly less stringent.

Specifically, a single new metabolite in the combined model is created by MetaMerge to represent each (matched) class of metabolites from the individual models, according to the matching matrix. Each reaction is then written using the new metabolites, and identical reactions are joined into a single reaction, treating the enzymes or enzyme combinations necessary to catalyze them as isozymes. This approach for combining models ensures that mass-balance constraints on each metabolite in the combined model are no more stringent than the corresponding constraints in the original model, because identifying two metabolites is equivalent to adding the corresponding constraints, and any flux vector satisfying both constraints satisfies their sum.

### Generating a combined model for M. tuberculosis

**Figure 2:**
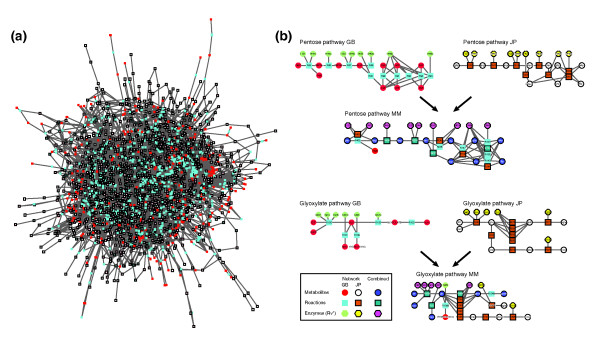
**The combined metabolic network for *M. tuberculosis***. **(a) **The combined model. Shown is the combined model for *M. tuberculosis *generated by MetaMerge. The network was laid out with Cytoscape [34] and 12 isolated reactions were removed from the final figure. The color scheme is identical to that of Figure 1, with the Beste *et al*. model [13] used as model 1 and the Jamshidi and Palsson model [14] used as model 2. **(b) **Example pathways in the original and combined models. Shown are the pentose phosphate and glyoxylate metabolism pathways in the original models (model 1, labeled GB [13], and model 2, labeled JP [14]) and the combined model. The enzymes catalyzing each reaction are included as a top layer, and their names are shortened by removing Rv. Circles indicate metabolites (red, model 1; white, model 2; pink, combined); squares indicate reactions (turquoise, model 1; brown, model 2; dark green, combined); octagons indicate enzymes (light green, model 1; yellow, model 2; magenta, combined). The subnetworks were laid out with Cytoscape [34] and several currency metabolites were removed for visual clarity.

We applied MetaMerge to the two *M. tuberculosis *models [13,14] and generated one combined model (Figure 2, Table 1). We considered three criteria to evaluate the quality of the combined model: (1) scope, reflected by the number of reactions, metabolites and enzymes captured in the model; (2) functionality, reflected by the number of reactions that can be reached by flux in any (at least one) steady state condition; and (3) relevance, reflected by the correspondence between the model's predictions of gene essentiality and available experimental data.

**Table 1:** Statistics for the three models

Number of	**Model 1 **[13]	**Model 2 **[14]	Model 3 (combined)
Genes	743	661	917
Reactions	873	973	1,400
Cytosolic reactions	741	837	1,207
Exchange reactions	132	100	193
Reversible reactions	187	254	180
Irreversible reactions	686	683	1,020
Metabolites	753	825	1,017
Internal metabolites	652	740	880
External metabolites	101	85	137

The combined model has a substantially broader scope than the two original models. It contains 1,400 reactions (349 from both models, 545 from model 1 only, 506 from model 2 only), with 1,017 metabolites (488 from both models, 276 from model 1 only, 253 from model 2 only) and 917 genes (487 from both models, 256 from model 1 only, 174 from model 2 only). Overall, 70% of the genes and metabolites are matched between the two models, but only 45% of the reactions are matched. This is due to the fact that we only considered two reactions to be identical if they agree completely in their metabolites according to the matching matrix (except for the currency metabolites H and H_2_O), as well as in their stoichiometric coefficients. However, another reason for the relatively low number of reactions that were matched between the two models appears to be the differential coverage of a number of important pathways by the two reconstructions.

There are 29 pairs of very similar reactions in the combined model (for a total of 58 reactions, or 4% of the combined set of reactions), where two reactions are considered to be very similar if they come from different original models and differ by only one metabolite (Additional file 1). In these cases, we kept both reactions in order not to prefer one model over the other. Given the different literature sources used in preparing the two reconstructions, it would require special expertise beyond the scope of our approach to decide which of the two reactions should be used as the correct one. Furthermore, keeping both reactions is necessary to ensure that the functionality of both initial models is preserved in the combined model, and indeed, the number of blocked reactions increased while the predictive power decreased when an arbitrary choice was made from each pair (data not shown). Finally, we intend the combined model to serve as complete a knowledge repository as possible, even at the cost of containing the same reaction in conflicting ways. MetaMerge can flag such conflicts, which can be subsequently resolved by a consensus of experts, as was recently done for yeast [17].

### The combined model reduces the number of non-functional reactions

One of the main challenges in reconstructed networks is the presence of 'blocked' reactions that can only have zero flux and are thus 'non-functional' at steady-state. A reaction can be blocked due to the topology of the network, its stoichiometry, or thermodynamic (irreversibility) constraints (Materials and methods). In general, a reaction is blocked if the linear program that constrains it to carry one unit of flux is infeasible.

We reasoned that combining the two models might provide the particularly important benefit of 'unblocking' some of the reactions that were blocked in the individual models, since previous constraints may be relaxed by additional reactions in the combined model and cannot become more stringent.

Indeed, in the combined model, the total number of blocked reactions decreases by 55%, from 246 to 109 (Table 2). In particular, 16 of 47 blocked reactions from model 1 and 118 of 199 from model 2 become unblocked in the combined model. An example reaction from model 1 that gets unblocked in the combined model is the transformation of p-hydroxyphenylpyruvate into homogentisate, a reaction specific to *M. tuberculosis *[8]. An example reaction from model 2 that gets unblocked is the transformation of nicotinamide into nicotinate, catalyzed by the gene product of *pncA *(Rv2043c), which is required for activation of the antitubercular drug pyrazinamide [18]. As discussed above, no new blocked reactions should appear. On the other hand, the fact that many blocked reactions become unblocked indicates the added synergy and points to the value of model integration.

**Table 2:** Blocked reactions in the three models

Reactions blocked due to	**Model 1 **[13]	**Model 2 **[14]	Model 3
Topology	20	88	68
Stoichiometry	3	6	1
Thermodynamics	24	105	40
Any of the above	47	199	109

### The combined model has a modestly increased ability to positively predict gene essentiality

We next examined the ability of our combined model to assess gene essentiality by comparing its predictions to those determined experimentally using transposon site hybridization (TraSH), a negative genetic selection method [19]. This essential gene list is the most commonly referenced set of *in vitro *essential genes for *M. tuberculosis *strain H37Rv. The TraSH data were obtained by growing H37Rv on Middlebrook medium 7H10 supplemented with glycerol and OADC (oleic acid, albumin, dextrose and catalase).

We approximated these conditions as closely as possible by simulating the *in vitro *growth of *M. tuberculosis *on Middlebrook medium 7H10, supplemented with glycerol [19]. To this end, we enabled all the reactions corresponding to the import of elements of the Middlebrook medium, as previously done [13,14]: ammonium, biotin, calcium, chloride, citrate, ferric iron, L-glutamic acid, phosphate, potassium, sodium, and sulfate, with the addition of glycerol. Additionally, we added the following nutrients for growth from model 1 [13]: carbon dioxide, molybdenum, nitrogen dioxide, and oxygen. For model 2 [14], the added nutrients were carbonic acid, copper, magnesium and oxygen. Notably, despite the importance attributed to fatty acid metabolism in *M. tuberculosis*, we did not include OADC (particularly the oleic acid component) in the computational medium's composition because oleic acid was not present in the original models. This limitation is also present in all previous studies [13,14].

We next predicted gene essentiality in the combined model and its two constituent models. We eliminated the enzymes one at a time from the model and tested the impact on the model's ability to exhibit flux through the biomass reaction, as an indicator of growth (Materials and methods). For the combined model and for model 1, we used the *in vitro *biomass composition as an indicator of growth. For model 2, we attempted to use the reduced biomass composition. However, the model was unable to produce any biomass on the medium described above when analyzed with any of six different linear programming solvers [20], whether cofactors were included or not. We therefore used only the biomass composition for model 1 and the combined model in all of our *in silico *experiments.

**Figure 3:**
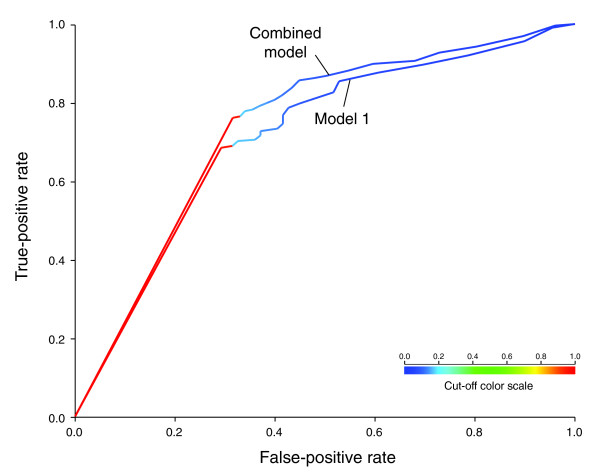
**Essentiality predictions in model I and in the combined model**. Shown are receiver operating characteristic curves for the correctly predicted fraction of gene essentiality (y-axis) based on model 1 and the combined model when the essentiality threshold for the TraSH experiment [19] is allowed to vary between 0 and 0.2 in increments of 0.002 (x-axis).

We compared gene essentiality from the TraSH experiments for model 1 and the combined model (Figure 3). Because the gene essentiality is determined as the absence of clones containing transposon insertion in essential genes, as detected by hybridization signal on a microarray, different thresholds (ratio of signal from a transposon insertion to genomic DNA) can be set for calling whether a gene is putatively essential or not. Thus, we chose to compare our model to TraSH prediction of essentiality using two different thresholds for calling a gene '*in vitro *essential' (Materials and methods). For each of the models, we considered a gene to be essential only when setting the flux through the corresponding reaction to 0 results in no biomass production. This allowed us to avoid imposing an arbitrary cutoff on the growth rate, but gave results for model 1 that differ somewhat from those previously reported for this model [13].

Fewer genes were predicted to be essential for the combined model than for model 1 (89 and 203, respectively), as expected given the expanded scope and functionality of the combined model (Table 3). Furthermore, the combined model has a slightly higher positive predictive value than model 1 at both tested thresholds for *in vitro *essentiality (Materials and methods), with a positive predictive value of 71% (63 true positives/89) versus 68% (139 true positives/203) at a threshold of 0.2. The negative predictive value, on the other hand, is somewhat lower at both thresholds calculated (0.2 and 0.1), with a negative predictive value of 68% (436 true negatives/637) versus 75% (285 true negatives/381) at a threshold of 0.2. These results are consistent across different thresholds (Figure 3). The *P*-value for the null hypothesis that the method randomly decides the essentiality or non-essentiality of each reaction, given by the Fisher exact test, was less than 10^-10^ in all cases. The preference between a higher positive predictive value and a higher negative predictive value may depend on the application.

**Table 3:** Gene essentiality in models 1 and 3 at two threshold ratios (the highest percentage achieved for each prediction metric appears in bold)

	**Model 1 **[13]		Model 3	
		
Predictions in	Threshold 0.1	Threshold 0.2	Threshold 0.1	Threshold 0.2
True positive	106	139	49	63
False positive	97	64	40	26
True negative	325	285	508	436
False negative	56	96	129	201
Positive predictive value (%)	52	68	55	**71**
Negative predictive value (%)	**85**	75	80	68
Correct predictions (%)	74	73	**77**	69

### Detecting enzymes whose perturbation mimics the effect of known drugs

We finally attempted to use the combined model to identify enzymes whose perturbation would mimic the metabolic effect of inhibiting known targets of anti-tubercular drugs. Given a known target enzyme, we found additional enzymes whose inhibition could have the same effect as a given drug by identifying all other reactions in the network whose flux is directly proportional to that of the reactions catalyzed by the drug target enzyme (Materials and methods). Briefly, all the reactions in a metabolic network can be divided into reaction subsets (also called 'enzyme subsets' [21]), such that all reactions in each subset always have pairwise proportional fluxes at steady-state. In particular, if one of them is blocked and has zero flux, all the other ones are blocked as well. We reasoned that if inhibition of the drug target has a desired effect, then inhibition of another enzyme from the same subset may have a similar effect on cellular metabolism.

We focused on comparison to the targets for two first-line antitubercular drugs, ethambutol (ETH) and isoniazid (INH) [18]. Ethambutol inhibits arabinogalactan synthesis, which is required for cell wall biosynthesis, predominantly through inhibition of EmbB (possibly also through EmbA), and inhibits lipoarabinomannin synthesis through inhibition of EmbC [22,23]. Isoniazid also inhibits cell wall synthesis after activation by the catalase KatG, resulting in an isoniazid-NAD adduct that inhibits InhA, an enoyl-acyl carrier protein reductase required for mycolic acid synthesis.

We first applied our approach to the proposed drug targets of ethambutol, the enzymes Rv3793 (embC), Rv3794 (embA) and Rv3795 (embB). We found three reactions catalyzed by these enzymes in the combined model. By examining the reaction subsets to which these belong, we found 29 additional enzymes whose inhibition is predicted to yield similar effects to those of known ethambutol targets (Additional file 2). Interestingly, two of these enzymes, Rv2051c (Ppm1) and Rv2611c, are also involved in the biosynthesis of lipoarabinomannan [24], though only one of them, Rv2611c, is predicted to be essential by TraSH. Thus, the use of the combined model successfully identified enzymes that function in the same metabolic pathway as EmbC. While most of the enzymes that were identified are not required for synthesis of arabinogalactan or lipoarabinomannan, enzymes that have some role in lipid and cell wall biosynthesis comprise the largest class. Finally, genes in many important metabolic pathways, including siderophore biosynthesis and cofactor biosynthesis, were also identified in this subset. The fact that many of the identified enzymes are not predicted to be essential by TraSH may be indicative of the limited predictive power of the combined model. It also highlights a general limitation of constraint-based metabolic network models, which is that reactions in an enzyme subset with an essential reaction are also predicted to be essential.

**Figure 4:**
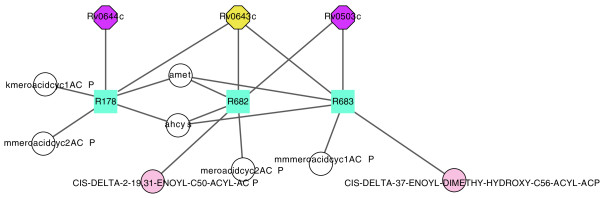
**Enzymes predicted to have similar metabolic impact to that of isoniazid targets**. Shown are the enzymes in the combined model identified to be in an enzyme subset with the targets of isoniazid, as well as the reactions for which these enzymes are essential. Circles indicate metabolites; squares indicate reactions; octagons indicate enzymes. The subnetwork was laid out with Cytoscape [34] and several currency metabolites were removed.

Similarly, when we applied this approach to the isoniazid target enzyme Rv1484 (inhA), we found 14 reactions disabled by isoniazid, and two enzymes, Rv0503c (CmaA2) and Rv0643c (MmaA3), that are predicted to have a similar metabolic effect (Figure 4, Table 4). CmaA2 and MmaA3 are components of the mycolic acid biosynthetic pathway of which InhA is a critical component. While detecting components of mycolic acid biosynthesis is interesting, CmaA1 and MmaA3 are not essential for the production of mycolic acids, but rather for specific modifications of the alkyl side chain of the mycolic acid, and these enzymes are not essential for *M. tuberculosis *growth.

**Table 4:** Metabolite features extracted and used by MetaMerge

Feature name	**Model 1 **[13]**(652 species)**	**Model 2 **[14]**(740 species)**
Official name	628	740
Abbreviation	652	740
IUPAC name	365	475
CAS number	227	297
Biocyc ID	340	463
KEGG ID	293	386
Chemical formula	318	740

## Conclusions

Here we presented MetaMerge, a method for semi-automatically combining two metabolic networks into a single network. The combined network preserves the full metabolic capabilities of the individual networks, while providing the possibility of synergistic interactions that lead to novel capabilities. With expert input, the resulting model can be further refined, for instance, by resolving the discrepancies between pairs of similar reactions (one from each of the original networks). In that case, MetaMerge can greatly reduce the time required to build a consensus model. Additionally, MetaMerge could be used to reconcile two networks corresponding to different, but closely related, organisms, although the result may or may not correspond to that of the metabolic network of their closest ancestor. MetaMerge may be best used in combination with a metabolic network alignment algorithm in order to gain evolutionary insights from the comparison of the networks for closely related organisms.

We applied MetaMerge to two existing network models for *M. tuberculosis *and used three criteria to assess the success of the combined model. The combined model provides better coverage of the space of metabolic reactions, has fewer blocked reactions (and hence significantly more effectively available reactions), and produces slightly better predictions of gene essentiality in terms of the positive predictive value (but slightly worse in terms of the negative predictive value). However, both the original and the combined model still do not capture the full range of gene essentiality information. The discrepancies between the model's prediction and experimental data can suggest areas where knowledge in the model should be improved, such as potentially missing reactions, missing gene-protein-reaction associations, or unnecessary restrictions on reaction directionality.

Finally, we used the combined model to suggest 31 genes whose inhibition is predicted to result in an effect similar to that of exposure to two known antitubercular drugs, many of which are within similar functional pathways. Notably, a similar, more stringent criterion, previously used by [14], is based on identifying enzymes that catalyze 'hard-coupled reactions': pairs of reactions uniquely producing and uniquely consuming a given metabolite. Hard-coupled reactions always belong to the same reaction subset, but reaction subsets cannot always be obtained from hard-coupled reactions. Indeed, when we repeated the same analysis using hard-coupled reactions instead of reaction subsets, we only found 11 of the 31 enzymes (Table 4).

MetaMerge in its current version results in a combined network that preserves the full metabolic capabilities of the individual networks while providing the possibility of synergistic interactions that lead to novel capabilities. If expertise is available, the resulting model can be further refined, for instance, by resolving the discrepancies between pairs of similar reactions (one from each of the original networks). In that case, MetaMerge can greatly reduce the time required to build a consensus model.

One of the main features of the MetaMerge algorithm is its ability to extract features of entities (metabolites, reactions and genes) present in a metabolic model in an automatic way by querying appropriate databases. Even for richly annotated models, this can be an important way of updating the annotation given that new biological information becomes available almost every day. It is conceivable that automated metabolic network reconstruction will become a possibility in the future, and a tool such as MetaMerge will then be crucial to gathering available information on organisms' metabolism, unifying information from disparate sources into a single model, and providing a natural starting point for metabolic network analysis.

## Materials and methods

### The MetaMerge method

Additional file 3 contains a step-by-step transcript of a Python session culminating in the creation of a combined *M. tuberculosis *model from the two original models and the production of an SBML (Systems Biology Markup Language) file containing the combined model. It precisely follows the stages that we outline here. We also provide the required scripts and input files as Additional file 4.

#### Stage 1: model parsing and feature preparation

We parsed the models using the *ModelParsing.py *script contained in Additional file 4. The parser detected several typos and inconsistencies, detailed in Additional file 5, which we corrected. We further converted the models [13,14] into version 2 level 4 of SBML [25], the current standard for metabolic networks. These models are provided as Additional files 6 and 7, respectively, and they have also been uploaded to the BioModels database [26].

The following features were used to compare metabolites: abbreviation, official name, CAS number [16], International Union of Pure and Applied Chemistry (IUPAC) name [27], BioCyc identifier [28], KEGG identifier [8], molecular formula (Table 4). We extracted the CAS numbers and IUPAC names semi-automatically because no freely accessible website had a URL structure that could be built explicitly based on the metabolite name. The BioCyc and KEGG identifiers were used to retrieve the CAS number and IUPAC name for metabolites for which they had not been found using the semi-automatic web search. To match official names, we used fuzzy string matching with the default cutoff of 0.6, implemented in Python [29] as the *get_close_matches *method in the *difflib *module. We considered only perfect matches for the remaining features.

The following features were used to compare reactions: reaction name, pathway name, gene name, protein name, enzyme name (Table 5). Unlike for metabolites, all of the reaction features were available directly from the complete models. However, to retrieve enzyme names from their EC numbers [30], we used an automatic search on the ExPASy Proteomics Server [31], implemented with the *urlopen *method in the *urllib *module in Python [29].

**Table 5:** Reaction features extracted and used by MetaMerge

Feature name	**Model 1 **[13]**(873 reactions)**	**Model 2 **[14]**(937 reactions)**
Pathway name	860	936
Reaction name	847	936
Enzyme name	562	936
Gene name	873	936
Protein name	538	720

#### Stage 2: initial matching of reactions and metabolites

We created two score matrices, *M *and *N*, using the features described immediately above. The features were each given a score of 1, although the code allows for user-defined unequal weights to be provided as well. The various names were considered to be a match if they contained at least one non-function word in common (at least two for the case of protein names and reaction names), and we only considered perfect matches for the remaining features. *M_ij_*(respectively *N_ij_*) is the total score of the matching features for the pair of metabolites (respectively reactions) *i *and *j*. We note that additional unique annotations, such as SMILES or InChi strings for metabolites, would make the reconciliation process less ambiguous, and therefore less error-prone for MetaMerge. We highly recommend their use in annotating genome-scale metabolic network reconstructions.

We also created two binary matching matrices, *M^B^*and *N^B^*, initialized to contain a 0 in every position. The entry *M^B^_ij_*is 0 if the two metabolites have not been matched and 1 if they have been matched. If the algorithm is being used interactively (with input from the user), these matrices become ternary: an entry of 1 means that the match has been accepted by the user, and -1, rejected, while an entry of 0 means that the match has never been proposed. The matrices *M^B^*and *N^B^*are then used during the iterative stage to keep track of matching decisions in the previous iterations.

To initialize a matching of reactions and metabolites, we used the pairs of reactions (*i*,*j*) with a total score *N_ij_*above a cutoff of 3. Subsequently, the highest-scoring pairing of the metabolites was computed for each of the reaction pairs in the matching using a greedy algorithm. This algorithm identifies the metabolites *k *in reaction *i *and *l *in reaction *j *with the largest total score *M_kl_*, matches these to one another, and then repeats the process with the remaining metabolites until all the metabolites in one of the reactions have been matched or all remaining pairs of metabolites have a total score of 0.

Although it would be possible to use an algorithm for maximum-weight bipartite matching instead of the greedy algorithm, examining multiple pairs of reactions did not reveal any example where the results would have been different from those of the greedy algorithm. In fact, very few metabolites in any pair of reactions have more than one possible match. Since a maximum-weight bipartite matching algorithm would have required users to download and install an extra Python library, we decided that this additional overhead would not be justified.

#### Stage 3: optional interactions with the user

The current set of newly matched reactions can be presented to the user for confirmation. The confirmation proceeds in two steps. First, the user confirms that the reactions should be matched to each other. Second, the user checks the matching between the metabolites in these two reactions.

We implemented several features in the user interface. The first set of features comprises inspection options, by which the user could see not just the score for a given pair of reactions or metabolites, but also their complete sets of features side-by-side, as well as any decisions about the pair that had been made previously. The second set of features comprises browsing options, which allow the user to go forward and backward in the list of matched reactions. The third set of features comprises confirmation options, which allow the user to accept or reject a proposed match of reactions or metabolites, as well as to enter their own match in either text format or using the numbers displayed next to each of the metabolites.

To speed up the confirmation process, the user also has the option of accepting all the proposed matches between the metabolites of two reactions. A metabolite could also be matched to 'nothing' if it was present in one reaction, but not in the other. In addition, when two internal metabolites are matched, the user is asked whether the corresponding external metabolites (if both exist) should be matched.

Although these interactions could, in principle, be bypassed entirely, we found that they were helpful in steering the algorithm away from possible mistakes in the matching process. For instance, several close matches with similar scores were sometimes available for a given reaction, but only one of them was the correct match, and this could only be detected by using the browsing feature of the interface. Furthermore, the possibility of seeing previous decisions on putative metabolite matches helps to maintain overall consistency in the matching, which prevents violations of transitivity (as discussed below).

#### Stage 4: extension of the current matching

During the matching of metabolites in matched reactions, new pairs of matching metabolites are usually found. If that is the case, the extension algorithm finds all the pairs of reactions that have not yet been matched, and whose metabolites could be matched almost perfectly (that is, all but one metabolite in each reaction can either be matched to one another or to nothing, based on previous confirmations). Convergence occurs when no further extension of the current matching is possible.

#### Stage 5: transitivity and non-transformability

In the postprocessing phase of the algorithm, the matching of the metabolites is cleaned up. This phase is currently performed in a semi-automatic way, although it might be possible to automate it completely. First, transitivity of the metabolite matching needs to be ensured. A matching matrix is said to be transitive if it can be perfectly covered by rectangles. In graph-theoretic terms, this is equivalent to saying that the bipartite graph formed by the matching is a disjoint union of bicliques [32]. The transitivity is easily checked by greedily covering the matching matrix with rectangles and seeing if any of them overlap. If so, the overlapping pairs of rectangles are presented to the user to decide how to remedy the problem.

Second, non-transformability of the metabolite matching needs to be ensured as well. A transitive matching matrix is said to be non-transformable if there is no rectangle in its covering that contains two metabolites from the same network that participate on different sides of a reaction. This will lead to the undesirable behavior of metabolites canceling out on either side of a reaction in the combined network.

A third condition that is not strictly necessary, but which makes the merging process a lot easier and cleaner, is compartmentalization. In the context of a model with only two compartments (the cell and the extracellular space) this simply means that no internal metabolite in one network is matched to an external metabolite in the other network. In the case of more complex models, such as the eight-compartment yeast model [10], this would mean that any pair of matched metabolites must belong to the same compartment.

#### Stage 6: creation of the combined network

In order to create the combined network, we combined each group of metabolites corresponding to a rectangle in the matching matrix (equivalently, a biclique in the matching graph) into a single new metabolite. Optionally, we allow for some of the metabolites that were frequently matched to 'nothing' to be deleted from the combined network. In the case of the two *M. tuberculosis *networks considered in this work, we deleted only protons (H) and water molecules (H_2_O). Each reaction is then rewritten using the new metabolites. This results in many identical reactions, which are considered to be isozymes of one another. The combined network contains only one reaction from each group of isozymes. To determine the reversibility of a reaction in the combined network, we examine the reactions from the original networks that are represented by it. If at least one of them is reversible or if two of them are oppositely directed, the new reaction is taken to be reversible; otherwise, it is taken to be irreversible. The network created by combining models 1 and 2 [13,14] is written out in SBML format [25] in Additional file 8.

### Identification of blocked reactions

For each reaction, we determined whether it is able to sustain a nonzero flux at steady-state, and if not, whether this is due to the topology, the stoichiometry or the thermodynamics of the model. Reaction *i *is said to be topologically blocked if it contains a unique internal metabolite (not present in any other reaction), stoichiometrically blocked if the mass balance condition *Sv *= 0 implies *v_i_*= 0, and thermodynamically blocked if the mass balance condition together with the irreversibility conditions implies *v_i_*= 0. In each case, we choose the simplest possible cause for the blockage. This analysis was performed using the MONGOOSE toolbox [33], described in Additional file 9.

### Construction of a gold standard for gene essentiality

To construct our gold standard for gene essentiality, we used results of the TraSH experiment [19] and followed the methodology of Beste *et al*. [13]. We tested different ratios of the microarray hybridization signal obtained from labeled insertion sites in a saturated transposon mutant library compared with a control of labeled genomic DNA. We used both the threshold 0.2 as in the original experiment, as well as the more stringent threshold 0.1, for deciding the essentiality of a gene based on the results of the TraSH experiments.

### Prediction of essential genes

For each reaction, we assembled the genes required to catalyze it in conjunctive normal form (OR of ANDs). From this information, we compiled a list of all the reactions disabled by the knockout of each gene. Subsequently, we constrained these reactions to have a flux of 0 and determined that the gene is essential if the resulting metabolic network was unable to exhibit growth, and non-essential otherwise. This analysis was performed using MONGOOSE [33].

### Determination of reaction subsets

To find all the reaction subsets, we first identify the blocked reactions, delete them from the stoichiometric matrix *S *of the network, and then compute the nullspace matrix *K *and identify sets of proportional columns. Two reactions, *i *and *j*, in a metabolic network with stoichiometric matrix *S *are said to be in a reaction subset if there exists a constant *κ *≠ 0 such that *Sv *= 0 implies *v*_i _= *κv_j_*. As explained in Additional file 8, if all blocked reactions are deleted from *S*, then all reaction subsets can be identified from the nullspace matrix *K *of *S*, regardless of the irreversibility of any of the reactions. In the combined network for *M. tuberculosis*, the size of such subsets varies between 2 and 38 reactions, and in fact, the largest subset with 38 reactions contains a reaction that is disabled by ethambutol.

## Abbreviations

CAS: Chemical Abstracts Service; IUPAC: International Union of Pure and Applied Chemistry; KEGG: Kyoto Encyclopedia of Genes and Genomes; OADC: oleic acid: albumin: dextrose and catalase; SBML: Systems Biology Markup Language; TraSH: transposon site hybridization.

## Authors' contributions

LC proposed the algorithm, carried out the analyses, and drafted the manuscript. SS and DH controlled the quality of the combined model and suggested the computational experiments for validating it. AR suggested the approach and helped devise a strategy for creating the combined model. BB guided the design of the study and suggested improvements to the MetaMerge algorithm. All authors helped revise the manuscript and approved the content of the final version.

## Supplementary Material

Additional file 1 **Reactions in models 1 and 2 differing by at most one metabolite**. There are 29 pairs of reactions (each pair consisting of one reaction from model 1 and one reaction from model 2) that differ by at most one metabolite. These reactions are given in the representation used in the original models. Each pair is followed by a line of dashes. Click here for file

Additional file 2 **Enzymes predicted to have similar metabolic impact to that of known drug targets**. There are 29 enzymes listed for ethambutol and 2 enzymes listed for isoniazid. Each enzyme is given with its gene ID and name, the set of reactions it is essential for in the initial models (uppercase metabolite names for [13], lowercase for [14]), its function, and its category. If a reaction for which an enzyme is essential is common to both models, only one model is chosen to represent it. Click here for file

Additional file 3 **A sample Python session yielding a combined *M. tuberculosis *model**. This file contains the step-by-step transcript of a Python session culminating in the creation of a combined *M. tuberculosis *model from the two original models and the production of an SBML file containing the combined model. Click here for file

Additional file 4 **The full code of the MetaMerge algorithm implemented in Python**. The code is divided into 14 modules, each of which contains multiple functions, as follows: *ClassDefinitions.py*, the code for defining all the formats used by MetaMerge internally; *FeatureMatching.py*, the code for matching species, reactions based on the available features; *FeaturePreparation.py*, the code for extracting metabolite and reaction features from text; *GeneProcessing.py*, the code for processing gene information; *MatchProcessing.py*, the code for processing the metabolite and reaction matching matrices; *MetaboliteMatching.py*, the code for generating and processing closely matching metabolites; *MetaMerge.py*, an initializer for the other modules required by the MetaMerge algorithm; *MetaMergeCore.py*, the user interface of MetaMerge for preparing the matching matrices; *ModelParsing.py*, the user interface for parsing a metabolic model in Excel or SBML format; *NetworkMerging.py*, the code for merging two networks based on their matching matrices; *OutputProcessing.py*, the code for processing the output of the MetaMerge algorithm; *ReactionMatching.py*, the code for generating and processing closely matching reactions; *Unrelated.py*, the code for analyzing a metabolic network, not directly related to MetaMerge; *Utilities.py*, the code of miscellaneous auxiliary functions used by the MetaMerge algorithm. Additionally, the zipped directory contains a shelve file called *Mappings *with KEGG and Biocyc identifiers for the metabolites in both *M. tuberculosis *models extracted by Jeremy Zucker, and the cleaned-up and extended Excel files *Mycobacterium tuberculosis 1.xls *and *Mycobacterium tuberculosis 2.xls *for models 1 [13] and 2 [14], respectively, containing additional annotation contributed by Marina Druz. Click here for file

Additional file 5 **A list of errors corrected in the original *M. tuberculosis *models**. This file contains a list of errors detected in the original *M. tuberculosis *models [13,14] and corrected in the Excel files contained in Additional file 4. Most of these are typographical errors, but some are due to inconsistent notations in different parts of the original Excel files. Click here for file

Additional file 6 **Model 1 (Beste *et al*. **[13]**) in SBML**. The model contains 873 reactions and 753 metabolites. Each reaction is annotated with lower and upper bounds on its flux, the EC numbers for the enzymes catalyzing it, the Boolean expression containing the genes it requires, the name and chemical equation of the reaction, and the pathway to which it belongs, whenever these are known. Each metabolite is annotated with its abbreviation, official name, molecular formula, IUPAC name, CAS number, and BioCyc and KEGG database identifiers, whenever these are known. Click here for file

Additional file 7 **Model 2 (Jamshidi and Palsson **[14]**) in SBML**. The model contains 937 reactions and 825 metabolites. Each reaction is annotated with its confidence score, the proteins needed to catalyze it, the Boolean expression containing the genes it requires, the name and chemical equation of the reaction, and the subsystem to which it belongs, whenever these are known. Each metabolite is annotated with its abbreviation, official name, molecular formula and charge, IUPAC name, CAS number, and BioCyc and KEGG database identifiers, whenever these are known. Click here for file

Additional file 8 **The combined *M*. *tuberculosis *model in SBML**. The model contains 1,400 reactions and 1,017 metabolites. Each reaction is annotated with the corresponding information from the reactions in the original models that it corresponds to. Each metabolite is similarly annotated with the corresponding information from the metabolites in the original models that it corresponds to. In case a reaction or metabolite in the combined model represents two or more reactions or metabolites from the same original model, the annotations are separated by 'or', while if those reactions or metabolites that come from different models are separated by 'OR'. Click here for file

Additional file 9 **The MONGOOSE toolbox**. The MONGOOSE (MetabOlic Network Growth OptimizatiOn Solved Exactly) toolbox [33] is a software suite we have developed, which gives certifiably correct results quickly and efficiently and is able to handle the largest metabolic model currently reconstructed. Its main features are the use of exact rational arithmetic, which avoids the risk of erroneous results due to rounding errors, as well as its ability to compress the metabolic network in order to speed up subsequent computations. This file describes in detail the algorithms underlying MONGOOSE [33]. Click here for file
