# Supplementary material for: MetaMerge: scaling up genome-scale metabolic reconstructions with application to Mycobacterium tuberculosis
Source: Genome Biol. 2012 Jan 31;13(1):r6. doi: 10.1186/gb-2012-13-1-r6 (PMC3488975; doi:10.1186/gb-2012-13-1-r6)
Supplement: Additional file 1 — Reactions in models 1 and 2 differing by at most one metabolite. There are 29 pairs of reactions (each pair consisting of one reaction from model 1 and one reaction from model 2) that differ by at most one metabolite. These reactions are given in the representation used in the original models. Each pair is followed by a line of dashes. [file gb-2012-13-1-r6-S1.DOC]

1.0 h + 1.0 lpam + 1.0 pyr <==> 1.0 adhlam + 1.0 co2

(1.0) LIPO + (1.0) PYR + (0.001) THI == (1.0) ADLIPO + (1.0) CO2

----------------------------------------

(1.0) F6P + (1.0) G3P + (0.001) THI == (1.0) E4P + (1.0) X5P

(1.0) E4P + (1.0) X5P + (0.001) THI == (1.0) F6P + (1.0) G3P

----------------------------------------

(1.0) F6P + (1.0) G3P + (0.001) THI == (1.0) E4P + (1.0) X5P

1.0 e4p + 1.0 xu5p-D <==> 1.0 f6p + 1.0 g3p

----------------------------------------

(1.0) G3P + (1.0) S7P + (0.001) THI == (1.0) R5P + (1.0) X5P

(1.0) R5P + (1.0) X5P + (0.001) THI == (1.0) G3P + (1.0) S7P

----------------------------------------

(1.0) G3P + (1.0) S7P + (0.001) THI == (1.0) R5P + (1.0) X5P

1.0 r5p + 1.0 xu5p-D <==> 1.0 g3p + 1.0 s7p

----------------------------------------

(1.0) E4P + (1.0) X5P + (0.001) THI == (1.0) F6P + (1.0) G3P

1.0 e4p + 1.0 xu5p-D <==> 1.0 f6p + 1.0 g3p

----------------------------------------

(1.0) R5P + (1.0) X5P + (0.001) THI == (1.0) G3P + (1.0) S7P

1.0 r5p + 1.0 xu5p-D <==> 1.0 g3p + 1.0 s7p

----------------------------------------

(1.0) O2 + (1.0) PRECORIN-3A == (1.0) PRECORIN-3B

1.0 h + 0.5 o2 + 1.0 pre3a <==> 1.0 pre3b

----------------------------------------

1.0 g6p + 1.0 nadp <==> 1.0 6pgl + 1.0 h + 1.0 nadph

(1.0) G6P + (1.0) NADP + (0.001) F420 == (1.0) D6PGL + (1.0) NADPH

----------------------------------------

(1.0) MYCOTHIOL-S-CONJUGATE == (0.999) NG-INS + (1.0) N-ACETYL-S-CONJUGATE

1.0 bmnmsh + 1.0 h2o <==> 1.0 acysbmn + 1.0 igam

----------------------------------------

(1.0) ADENOSYLCOBINAMIDE-GDP == (1.0) COB-I + (1.0) GMP

1.0 agdpcbi + 1.0 rdmbzi <==> 1.0 adocbl + 1.0 gmp + 1.0 h

----------------------------------------

(1.0) ADP + (1.0) O2 + (1.0) RTHIO == (1.0) DADP + (1.0) OTHIO

1.0 adp + 1.0 trdrd <==> 1.0 dadp + 1.0 h2o + 1.0 trdox

----------------------------------------

(1.0) 7-8-DIAMINONONANOATE + (1.0) ATP + (1.0) CO2 == (1.0) ADP + (1.0) DETHIOBIOTIN

1.0 atp + 1.0 co2 + 1.0 dann <==> 1.0 adp + 1.0 dtbt + 3.0 h + 1.0 pi

----------------------------------------

1.0 h + 2.0 pyr <==> 1.0 alac-S + 1.0 co2

(2.0) PYR + (0.001) THI == (1.0) ACLAC + (1.0) CO2

----------------------------------------

(1.0) ATP + (1.0) BIOTIN == (1.0) AMP + (1.0) BIOTIN-BCCP + (1.0) PPI

1.0 atp + 1.0 btn + 1.0 h <==> 1.0 btamp + 1.0 ppi

----------------------------------------

(1.0) CDP + (1.0) O2 + (1.0) RTHIO == (1.0) DCDP + (1.0) OTHIO

1.0 cdp + 1.0 trdrd <==> 1.0 dcdp + 1.0 h2o + 1.0 trdox

----------------------------------------

(1.0) UREA == (1.0) CO2 + (1.0) NH3

2.0 h + 1.0 h2o + 1.0 urea <==> 1.0 co2 + 2.0 nh4

----------------------------------------

(1.0) GDP + (1.0) O2 + (1.0) RTHIO == (1.0) DGDP + (1.0) OTHIO

1.0 gdp + 1.0 trdrd <==> 1.0 dgdp + 1.0 h2o + 1.0 trdox

----------------------------------------

(1.0) O2 + (1.0) RTHIO + (1.0) UDP == (1.0) DUDP + (1.0) OTHIO

1.0 trdrd + 1.0 udp <==> 1.0 dudp + 1.0 h2o + 1.0 trdox

----------------------------------------

(1.0) NADPH + (1.0) TRNA-GLU == (1.0) GLU1SEMIALD + (1.0) NADP

1.0 glutrna + 1.0 h + 1.0 nadph <==> 1.0 glu1sa + 1.0 nadp + 1.0 trnaglu

----------------------------------------

(1.0) RMMALONYLCOA + (0.001) COB-III == (1.0) SUCCOA

1.0 succoa <==> 1.0 mmcoa-R

----------------------------------------

(1.0) RMMALONYLCOA + (0.001) COB-III == (1.0) SUCCOA

(1.0) SUCCOA + (0.001) COB-III == (1.0) RMMALONYLCOA

----------------------------------------

1.0 succoa <==> 1.0 mmcoa-R

(1.0) SUCCOA + (0.001) COB-III == (1.0) RMMALONYLCOA

----------------------------------------

1.0 h + 1.0 nadph + 1.0 o2 + 1.0 uamr <==> 1.0 h2o + 1.0 nadp + 1.0 ugmr

(1.0) NADPH + (1.0) UDPNAM == (1.0) NADP + (1.0) UDPNGM

----------------------------------------

2.0 h + 1.0 no3 + 1.0 q8h2 <==> 2.0 h[e] + 1.0 h2o + 1.0 no2 + 1.0 q8

(1.0) MKH2 + (1.0) NO3 + (0.001) MOLYBDENUM-CO == (1.0) MK + (1.0) NO2 + (2.0) H

----------------------------------------

1.0 pep + 1.0 uacgam <==> 1.0 pi + 1.0 uaccg

(1.0) PEP + (1.0) UDPNAG == (1.0) UDPNAGPEE

----------------------------------------
