# Supplementary material for: MetaMerge: scaling up genome-scale metabolic reconstructions with application to Mycobacterium tuberculosis
Source: Genome Biol. 2012 Jan 31;13(1):r6. doi: 10.1186/gb-2012-13-1-r6 (PMC3488975; doi:10.1186/gb-2012-13-1-r6)
Supplement: Additional file 9 — The MONGOOSE toolbox. The MONGOOSE (MetabOlic Network Growth OptimizatiOn Solved Exactly) toolbox [33] is a software suite we have developed, which gives certifiably correct results quickly and efficiently and is able to handle the largest metabolic model currently reconstructed. Its main features are the use of exact rational arithmetic, which avoids the risk of erroneous results due to rounding errors, as well as its ability to compress the metabolic network in order to speed up subsequent computations. This file describes in detail the algorithms underlying MONGOOSE [33]. [file gb-2012-13-1-r6-S9.PDF]

The MONGOOSE Toolbox

January 12, 2012

1 Introduction

A number of tools for analyzing constraint-based metabolic models are available to the scientific community [1, 2, 3]. However, these tools have important limitations: some of them, based on floating-point arithmetic, may yield incorrect qualitative predictions, while others are too computationally demanding for large genome-scale metabolic models, and few are able to answer all the questions a researcher may ask. We address these limitations in the following way. First, we restate important questions in more tractable terms using new structural insights. Second, we incorporate procedures in exact arithmetic to prevent numerical inaccuracies. Finally, we propose an integrated pipeline for performing a complete qualitative analysis of constraint-based models. This analysis is facilitated by the MONGOOSE (MetabOlic Network GrOwth Optimization Solved Exactly) package we developed in-house.

2 Methods

This section explains the algorithms underlying the MONGOOSE toolbox, as well as some of its capabilities. Throughout this section, we use S to denote the stoichiometric matrix of the metabolic network. We remove the rows corresponding to external metabolites from S because they are generally not constrained to be balanced at steady-state.

2.1 Model definition

The system represented by the metabolic network is assumed to be at a quasi-steady-state, so that each internal metabolite is constrained to be balanced. The state of the system is represented by a mode (or flux vector) v, which specifies the number of times each reaction occurs in a given time period. The quasi-steady-state assumption implies that v is a mode of the system if and only if

$$Sv = 0 \text{ and } v_i \geq 0 \; \forall \, i \in \mathcal{I}, \tag{1}$$

where $\mathcal{I}$ is the set of reactions assumed to be irreversible.

2.2 Blocked reactions

Reaction i will be called *blocked* if conditions 1 imply that $v_i = 0$. We now identify three distinct reasons why a reaction could be blocked in a network.

The first reason is the *topology*, or connectivity, of the network. If reaction i involves a metabolite that is unique to it, the mass balance condition on this metabolite immediately implies that the flux through that reaction is 0. Additionally, if there is another reaction j that shares a unique metabolite with reaction i, it is also blocked as a consequence. These consequences can propagate through the network, sometimes in a dramatic way. When a reaction is blocked because of network topology in this way, we call it *topologically blocked.* All topologically blocked reactions can be identified by the following algorithm.

Algorithm1 [Topologically blocked reactions]
Input: a stoichiometric matrix S.
Output: the set Q of all topologically blocked reactions in S.
Initialization: $Q := \emptyset; d_j := |\{i|S_{i,j} \neq 0\}| \; \forall \; j$.
Iteration: **while** there exists j such that $d_j = 1$ **do**
$B := \{j|d_j = 1\}$
$Q_{\text{new}} := \{i|S_{i,j} \neq 0 \text{ for some } j \in B\}$
for $i \in Q_{\text{new}}$
for j such that $S_{i,j} \neq 0$
$d_j := d_j - 1$
$Q = Q \cup Q_{\text{new}}$
Termination: **return** Q.

The second reason is stoichiometry. Even when a reaction does not contain any unique metabolites, it is still possible that the stoichiometry of the network forces it to be blocked. This happens when $Sv = 0$ by itself (without any non-negativity constraints) implies $v_i = 0$. We call all such reactions *stoichiometrically blocked* because their blockage is caused by stoichiometry. It is possible, by performing a Gauss-Jordan elimination on S, to determine all such reactions. The reduced row-echelon form of S will contain e_i, the vector with 1 at position i and 0 elsewhere, as one of its rows if and only if e_i is in the rowspace of S. We restate this fact as an algorithm.

Algorithm2 [Stoichiometrically blocked reactions]
Input: a stoichiometric matrix S.
Output: the set Q of all stoichiometrically blocked reactions in S.
Algorithm: Compute R, the reduced row-echelon form of S; $Q := \{i|e_i \text{ a row of } R\}$.
Termination: **return** Q.

The third reason is thermodynamics. Even though a reaction may not blocked if only the mass-balance conditions are imposed on it, it may become blocked when the additional constraints $v_i \geq 0 \; \forall \; i \in \mathcal{I}$ are imposed. We call all such reactions *thermodynamically blocked* because their blockage is caused by thermodynamics. Note that all stoichiometrically blocked reactions are thermodynamically blocked, but not vice versa.

If our network has only irreversible reactions, Farkas' lemma shows that reaction i is thermodynamically blocked precisely when a vector $v \geq \mathbf{0}$ with

$v_i > 0$ is in the rowspace of S. In this way, one can see that a set X of reactions is thermodynamically blocked if and only if there is a nonnegative vector in the rowspace of S which has positive components in X. If X_1 and X_2 are two such subsets (not necessarily disjoint), then adding the corresponding vectors shows that $X_1 \cup X_2$ is also such a subset. Hence, we need to find the largest set of positive components (which we call "positive support") of a non-negative vector v in the rowspace of S, which is also the nullspace of the nullspace matrix K. This is done by the following algorithm.

Algorithm3 [Thermodynamically blocked reactions]
Input: a stoichiometric matrix S together with its nullspace matrix K.
Output: the largest positive support Q of a vector $x \geq \mathbf{0}$ in K's nullspace.
Initialization: $w := \mathbf{1}$ (the vector of all ones); $Q := \emptyset$; $z := 1$.
Iteration: **while** $z > 0$ **do**
Solve $z := \max \sum_i w_i x_i$ subject to $Kx = \mathbf{0}, \mathbf{0} \leq x \leq \mathbf{1}$.
$w_i := 0 \; \forall \; i \in R(x)$
$Q := Q \cup R(x)$.
Termination: **return** Q.

Now, while algorithm 1 is general, algorithms 2 and 3 apply only to the case of only reversible and only irreversible reactions, respectively. However, it turns out that they suffice to identify all the blocked reactions in the general case. The following algorithm shows how this can be done.

Algorithm4 [Removing all blocked reactions]
Input: a stoichiometric matrix S, the irreversible subset $\mathcal{I}$.
Output: S with all the blocked reactions removed.
Algorithm: $Q :=$ **Algorithm1**(S)
$S := S_{-Q}$ (S with the columns in Q removed)
$K :=$ **NullspaceMatrix**(S)
$Q :=$ **Algorithm3**$(K_{\mathcal{I}})$
$S := S_{-Q}$
$Q :=$ **Algorithm2**(S)
$S := S_{-Q}$
Termination: **return** S.

2.3 Enzyme subsets

An enzyme subset is defined as a set of reactions such that any steady-state fluxes in a set are in a fixed ratio. In particular, two reactions, say i and j, are part of the same enzyme subset if and only if there is a constant $\kappa \neq 0$ such that $v_i = \kappa v_j$ for all flux modes v. It was stated in [4] that most enzyme subsets can be identified by analyzing the nullspace matrix K and identifying proportional columns. Using the structure of constraint-based metabolic models we in fact proved that this approach can identify all the enzyme subsets. To construct the nullspace matrix K, a row-reduced echelon form of the stoichiometric matrix S is first computed in exact rational arithmetic.

2.4 Essential genes

A gene is called essential under given conditions if its deletion does not allow the organism to survive. In a metabolic model, a gene is predicted to be essential if disabling the reactions it is associated with blocks the biomass reaction. To test whether a gene g is essential, we check whether the biomass reaction can be active when equation (1) holds with $v_i = 0$ for every reaction i that is disabled by the deletion of g. Note that every reaction in the same enzyme subset as the biomass reaction is automatically essential, no matter what the conditions are.

3 Implementation

COBRA [1], one of the most popular packages for metabolic network analysis, uses floating-point calculations. Generally speaking, most linear-algebraic manipulations can be performed in a way that is *backward stable*, i.e. yielding the exact solution to a slightly perturbed version of the original problem [5]. The algorithms implemented in high-level programming languages are able to handle *rounding errors* fairly well. However, it turns out that backward stability is not sufficient for the analysis of metabolic networks.

All the stoichiometric matrices we deal with have rational coefficients. Most of the time, these coefficients are small integers. For a metabolic network represented by such a matrix, the answer to the question of whether a slightly perturbed version of the network can have a non-zero flux through a given reaction i yields no information about whether this is true for the actual network. For this reason we always perform our calculations in exact rational arithmetic.

All our algorithms are implemented in Python [6] because it is a high-level language that is freely available to the public and because it provides a flexible and versatile set of *modules*. Python's *fractions* module, available in versions 2.6 and higher, allows all the processing to be done in fractional arithmetic. However, to ensure backward compatibility we also implemented our own version of fractional arithmetic, which can be used with earlier versions of Python.

References

[1] **COBRA toolbox** [[http://gcrg.ucsd.edu/Downloads/Cobra_Toolbox]].

[2] **Metatool** [[http://pinguin.biologie.uni-jena.de/bioinformatik/networks/]].

[3] **CellNetAnalyzer** [[http://www.mpi-magdeburg.mpg.de/projects/cna/cna.html]].

[4] Gagneur J, Klamt S: **Computation of elementary modes: a unifying framework and the new binary approach**. *BMC Bioinformatics* 2004.

[5] Trefethen L, Bau D: *Numerical Linear Algebra.* Society for Industrial and Applied Mathematics 1997.

[6] **Python Software Foundation** [[http://www.python.org/psf]].
